# MONA – Interactive manipulation of molecule collections

**DOI:** 10.1186/1758-2946-5-38

**Published:** 2013-08-28

**Authors:** Matthias Hilbig, Sascha Urbaczek, Inken Groth, Stefan Heuser, Matthias Rarey

**Affiliations:** 1 Center for Bioinformatics (ZBH), University of Hamburg, Bundesstrasse 43, 20146 Hamburg, Germany; 2 Beiersdorf AG, Research Active Ingredients, Troplowitzstrasse 15, 22529 Hamburg, Germany; 3 Nuremberg Institute of Technology Georg Simon Ohm, Kesslerplatz 12, 90121 Nuremberg, Germany

## Abstract

Working with small‐molecule datasets is a routine task for cheminformaticians and chemists. The analysis and comparison of vendor catalogues and the compilation of promising candidates as starting points for screening campaigns are but a few very common applications. The workflows applied for this purpose usually consist of multiple basic cheminformatics tasks such as checking for duplicates or filtering by physico‐chemical properties. Pipelining tools allow to create and change such workflows without much effort, but usually do not support interventions once the pipeline has been started. In many contexts, however, the best suited workflow is not known in advance, thus making it necessary to take the results of the previous steps into consideration before proceeding.

To support intuition‐driven processing of compound collections, we developed MONA, an interactive tool that has been designed to prepare and visualize large small‐molecule datasets. Using an SQL database common cheminformatics tasks such as analysis and filtering can be performed interactively with various methods for visual support. Great care was taken in creating a simple, intuitive user interface which can be instantly used without any setup steps. MONA combines the interactivity of molecule database systems with the simplicity of pipelining tools, thus enabling the case‐to‐case application of chemistry expert knowledge. The current version is available free of charge for academic use and can be downloaded at http://www.zbh.uni‐hamburg.de/mona.

## Background

The compilation and preparation of small‐molecule datasets forms the core of virtually all cheminformatics applications. The careful selection of relevant compounds and the thorough processing of the associated data are essential in order to obtain meaningful results. Although the necessary steps for this process strongly depend on the respective context, there are nevertheless a number of common and recurring tasks. These include, among others, the removal of duplicates, filtering by physico‐chemical properties or substructure matching and the visual inspection of the respective compounds.

Workflow or pipelining tools support this recurrence by providing components or nodes corresponding to such common tasks. These nodes can be individually parameterized and combined in a pipeline, thus enabling the generation of a variety of customized workflows. The specification of these workflows is usually facilitated by a graphical interface. The most commonly used programs in the context of cheminformatics are Pipeline Pilot [1] and the open‐source alternative Knime [2] which have been compared in a recent review [3]. There are numerous further examples of scientific workflow systems described in the literature [4]. All these programs contain a certain number of predefined components and are extensible by allowing users to program their own modules. In addition to the flexibility concerning the specification of workflows, pipelining tools have the advantage that the processes are completely automated. This makes workflow processing the method of choice when all steps are known in advance and no intervention is necessary. Furthermore, there are usually only short setup times compared to the laborious installation and initialization of a server‐based molecular database system. Molecular databases, on the other hand, make it possible to compile datasets in a more interactive manner. Data needed for common cheminformatics tasks can be calculated in advance and stored in the database, resulting in noticeably reduced run times for data access. For most common database systems chemical cartridges exist which provide the functionality to import chemical data. Molecules are typically written to SQL tables in the form of line notations such as (U)SMILES [5] or InChI [6]. These unique topological identifiers are used to ensure the uniqueness of molecules or to rapidly find particular molecules in the database. It is possible to reduce run times for substructure searches by annotating common substructures in molecules and for similarity searches by using pre‐calculated fingerprints. Physico‐chemical properties can be stored in databases using indices to boost the run times of filter operations. Depending on the number and kind of pre‐calculated molecular descriptors, run times for setting up the databases can be quite large. Additionally, database systems often need to be installed on the respective operating system.

Here, we present MONA, a software tool aiming at combining the advantages of both approaches. In this way, the software enables a more interactive and intuitive approach to deal with large compound collections. In different validation procedures we show the internal consistency of all provided operations. Additionally we provide benchmarks showing that all provided operations are sufficiently fast for interactive use.

## Methods

**Figure 1:**
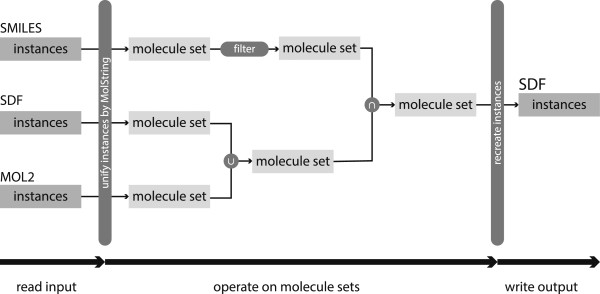
Schematic of a typical workflow using MONA.

Based upon the NAOMI framework [7], MONA allows to interactively prepare, inspect and convert small‐molecule datasets. The most important aspect of MONA is that the primary objects handled are molecules, not their occurrences in a particular dataset. During the import procedure, molecules are converted into a unique topological description, duplicates are automatically detected and stored as so‐called instances. A typical MONA workflow scheme is shown in Figure 1. To ensure high efficiency, MONA employs a relational SQL database for all operations on datasets. Furthermore, MONA’s architecture allows an efficient handling of molecule sets including their instant creation as well as classical set operations like union, intersection and difference.

The following sections describe the concepts behind MONA. This includes molecular representation and management by a relational database, performing operations on molecule sets, and rapid visualization of large compound collections.

### Molecules and instances

**Figure 2:**
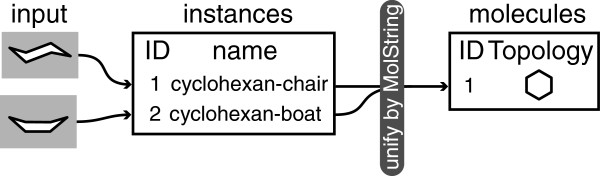
**Handling of instances in MONA.** Input structures with different coordinates but identical topology are assigned to the same molecule.

In the context of MONA the terms molecule and instance are used to distinguish between the actual compound and its occurrence in a dataset (see Figure 2). There can be multiple instances of the same molecule originating from different entries of input files. Depending on the context these instances can be interpreted as either conformations or duplicate entries. In order to reliably assign instances to their corresponding molecules, a canonical topological description is needed. MONA uses an internal string representation called MolString which serves two purposes. First and foremost it is used to efficiently rebuild the molecule as this is needed for particular operations as explained in the following sections. Furthermore, it is used as unique topological descriptor for the assignment of instances to molecules during registration. Molecules are serialized to and from the database, where each molecule and each instance is identified internally by an unique id called *Molecule Key* and *Instance Key* respectively.

Instances can be imported from common chemical file formats (SMILES, SDF, MOL2) using the NAOMI framework. The procedures for the consistent handling of these formats have been described in detail in [7]. If an entry consists of multiple disconnected components, currently solely the largest component is kept. Furthermore, it is possible to import small molecules from PDB files using the method described in [8]. In this case all components of the entry are imported. Additional data from SDF files is stored for each entry and can be recreated during export. Since the identification of molecules is based on a topological description, different tautomeric forms and protonation states are generally handled as separate entities. The same also applies to molecules with and without explicit specification of stereo descriptors. In order to customize the way molecules are assigned to instances, MONA offers different rules for the import of molecules. Depending on the context, molecules can be imported in a neutralized form, as canonized tautomer and without stereochemistry.

### Molecule sets

MONA allows to organize compounds in molecule sets. Molecule sets are collections of pair‐wise different molecules (not instances) which are used for all operations in MONA. As has been mentioned above, molecules are considered equal if and only if their canonical MolString representation is identical. We believe that this concept of molecular identity follows the basic understanding of chemists. Additionally, there are various technical reasons why sets of molecules are used rather than sets of instances. All available operations, such as filtering, manual selection and visualization, are based on molecular topology, so that there would not be any benefit from using sets of instances. Furthermore, some operations are based on the equality of the sets’ elements. Due to the additional data from the input format equality of instances is ambiguous at best, whereas it is well defined for molecules on the topological level. In the end, working with molecule sets is more efficient and the results from set operations can be intuitively understood.

Molecule sets are stored internally as lists of *Molecule Keys*. MONA is able to handle an arbitrary number by keeping these lists in a relational database. When exporting molecule sets to chemical file formats, molecules must be converted back to instances. As instances for a given molecule may come from different input files, it is necessary to choose which source should be used for output generation. For that purpose, a list of original molecule sources is kept in the database. Data associated with a molecule, such as names and coordinates, are then either taken from the first found instance or from all instances in the chosen data sources and eventually exported to the output file.

### Visualization of molecule sets

The analysis of the distribution of different physico‐chemical properties is a simple way to get a first impression of a molecule set. For that purpose MONA offers customizable histograms for a number of common physico‐chemical properties. It is also possible to include multiple sets in one histogram, which allows to compare their properties at a quick glance.

For further analysis, MONA offers a fast visualization of molecule sets using two‐dimensional structure diagrams. This provides a means to visually inspect large molecule collections and manually select molecules for the creation of smaller sets. MONA does not offer any type of three‐dimensional visualization which would only be needed to show differences between instances such as conformational variability. The necessary two‐dimensional coordinates are generated by a built‐in layout algorithm on the fly. In order to browse large molecule sets, the results of such calculations for the molecules must be available instantly. Even with a fast layout algorithm the pre‐calculation of coordinates for all molecules in a set would take a prohibitively long time. Fortunately, coordinates for all molecules are really never needed. By using a model‐view architecture and lazily calculating coordinates only when they are needed, browsing of molecule sets with hundred thousands of molecules becomes instantaneous. On modern hardware depictions of the few molecules a user can capture simultaneously on the computer screen appear without much latency. By intelligent multi‐threading, including the cancellation of coordinate calculations for molecules that are no longer visible, fast scrolling of large sets does not lead to congested threads.

### Operations on molecule sets

**Figure 3:**
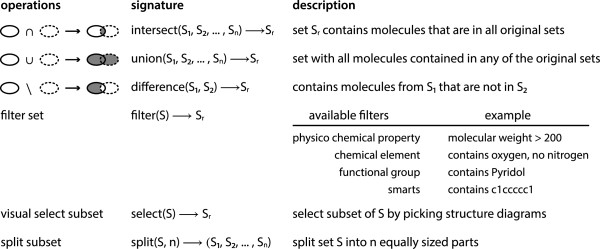
Supported operations in MONA.

In general, MONA operates on molecule sets and creates new sets as results (see Figure 3). All sets can be used in further operations resulting in a high degree of flexibility. The intention of the set concept is to enable the typical workflow of interactive processing, namely to browse, select, and store data iteratively. The common mathematical set operations (union, intersection and difference) work on multiple input sets and produce a single set as result. Since these operations are solely based on the evaluation of identities of the contained molecules, they can be realized directly by the database using SQL statements. Because molecule sets are internally handled as lists of *Molecule Keys* the respective operations can be carried out efficiently. Mathematical set operations produce results instantaneously even for large datasets, which makes them suitable for interactive use. For the same reasons, the splitting of molecule sets by various criteria is interactively possible.

### Filtering and visual selection

Both filtering and visual selection are operations on a single molecule set which generate a subset by excluding particular elements. The criterion for the exclusion is either a combination of molecular properties or manual selection. Filter chains for molecular properties are specified as a logical conjunction of elementary filters. Four elementary filter types are currently supported: (a) physico‐chemical properties, (b) chemical elements, (c) functional groups, and (d) SMARTS patterns.

The physico‐chemical properties comprise mostly topological descriptors such as the number of rings, molecular weight, and the topological surface area. This is extended by properties which can be derived from the chemical structure such as LogP [9]. Property filters always include or exclude a range of values the molecules must conform to. In contrast to that, substructure filters only ensure the presence or absence of a specific substructure in the molecules of the set. Chemical element filters are the most basic type of substructure filters. They are typically used to remove large classes of molecules such as halogenated compounds. Functional group filters allow the exclusion or inclusion of a set of common functional groups including both aromatic rings and acyclic structures. The number of groups and their types are currently predefined in MONA. If these should not be sufficient, SMARTS expressions can be used to handle any type of chemical patterns. Additionally, MONA allows to upload collections of SMARTS patterns and use them in a single query. The efficiency of the filtering operation strongly depends on the selected filter types. Property filters are fast since the values for molecules are pre‐calculated and stored in the database. These filters can therefore be realized by directly using database functionality. The same holds true for element and functional group filters. Both resort to pre‐calculated bitfields saved in the database. These are slower than the property filter as SQL databases do not support bitfield matches. SMARTS filters are the computationally most demanding types, since all molecules have to be rebuild from their MolString and tested against the SMARTS expression.

Elementary filters can be combined into complex queries which can be applied to any molecule set. In order to make filtering with criteria such as the Rule‐of‐Five for orally bioavailable molecules [10] possible, a tolerance can optionally be specified for a filter chain. This means that not all elementary filters need to match but only *m* of *n* filters, where *m* ≤ *n* can be arbitrarily chosen. Using tolerances has an impact on the speed of filtering operations. If *m* < *n* the filter process becomes slower, since the filter chain needs to be transformed into multiple database queries instead of one.

## MONA as application

MONA is a cross‐platform application, which can be started without prior installation as no setup of an external database system is required. Currently SQLite is used as underlying database backend for its simplicity in setup and administration. SQLite is connected via a regular SQL API such that any other relational database system could be used instead.

**Figure 4:**
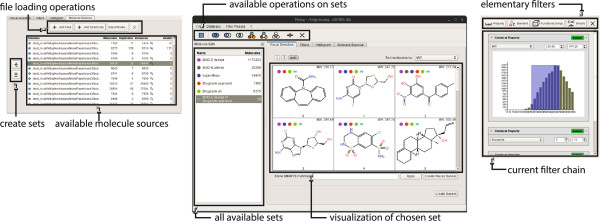
**MONA running on Linux.** Molecules are added from files via the file menu or the *Molecule Sources* tab shown on the left. 2D structure diagrams can be browsed in the *Visual Selection* tab shown in the middle, and filter chains are created using the *Filters* tab on the right.

The user interface consists of three different areas reflecting the functionality described in the previous sections. Imported molecule files are contained in the molecule sources view, from where molecule sets can be created at any time. The current molecule sets are shown in the list on the left side. They can be visualized in the respective views either as histograms or as a sortable table of structure diagrams. Operations for sets as described above are available in the toolbar or via the context menu. Filter chains can easily be build in the filter view (see Figure 4) using particular GUI elements for each type of elementary filter. Physico‐chemical property filters are created with the help of a histogram that shows the distribution of the selected property in the currently chosen set. Chemical elements in the element filter can be selected in a periodic table, and functional groups are specified using structure diagrams. SMARTS expressions are entered in text form, the syntax is checked while typing and wrong expressions are highlighted.

All operations run in separate threads, which is the basis of this responsive user interface. It maintains its performance even if more demanding tasks are running in the background. Created molecule sets can be saved persistently in the database and restored when opening the database again. Molecule sets can eventually be exported to one of the supported chemical file formats from the context menu.

## Results and discussion

The main focus of MONA are interactive scenarios where large molecule files need to be handled. To illustrate this further, three different workflows are described:

### Scenario 1: Preparing a molecule dataset for screening

**Figure 5:**
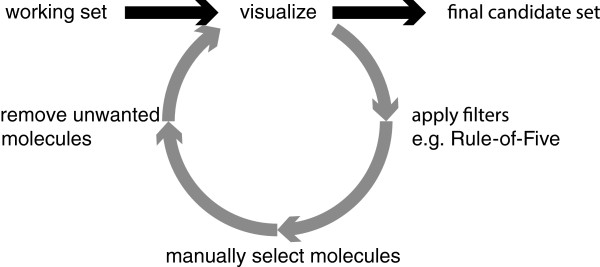
**Preparing a molecule dataset for virtual screening.** MONA allows to iteratively and interactively apply filtering steps to create suitable candidate sets.

The compilation of a set of molecules for a virtual or experimental screening is a very common task in cheminformatics. Starting with a large collection of compounds the preparation mainly consists of selecting a subset of molecules with suitable properties for the target to be addressed (see Figure 5). For this purpose various filters can be iteratively created and tested. A few common filters, e.g., the Rule‐of‐Five, are already predefined in MONA and can be used directly. In addition to the use of filters, molecules can also be selected manually using visual selection. The manual selection can often be facilitated by sorting the molecules according to a specific property. If the results of different filter runs are kept as sets, they can be compared to each other using set operations. Set operations can also be used to eliminate particular molecules (rather than substructures) from molecule sets. One can simply load a file containing unwanted compounds and subtract them from the current set. All steps can be iteratively applied after visual inspection of the remaining and the rejected molecules. For example, bounds related to physico‐chemical properties can be adapted on a case‐to‐case basis depending on the size of the remaining library. After finding the right combination of filters the final candidate set can be exported into an appropriate file format and used by another program. All data including 3D coordinates from instances previously read into the database are retained in this step.

### Scenario 2: Handling catalogs of molecules

**Figure 6:**
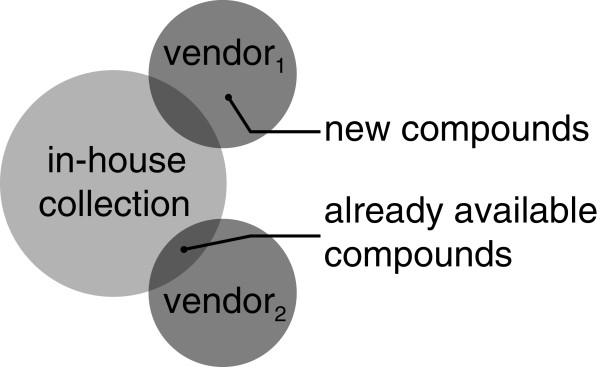
**Handling catalogs of molecules.** Set operations can be used to compare different compound collections by identifying molecules present in both.

The second scenario is taken from the field of compound management. Many vendors offer their compound catalogs in the form of chemical data files. These files can be used to compare the compound portfolio of the different vendors with each other or with an in‐house library (see Figure 6). This task is usually complicated by the fact that each vendor uses different standards for the representation of the respective compounds. When loading vendor catalogs as sets within MONA, different file formats and molecules across different vendors are automatically unified. Optionally, the user can decide to unify additional properties like the tautomeric state or the protonation. The resulting individual sets can be intersected with each other for comparison and evaluation. In this way either compounds offered by various vendors or substances that are uniquely supplied by one vendor can be easily identified. Furthermore, the sets can also be intersected with a current in‐house collection, so that potential additions may be identified. Vendor catalogs usually contain price information and order numbers for each compound. Exporting all instances for molecule sets preserves this information and allows to compare prices for all molecules in the exported set.

### Scenario 3: Verifying existing molecular databases

**Figure 7:**
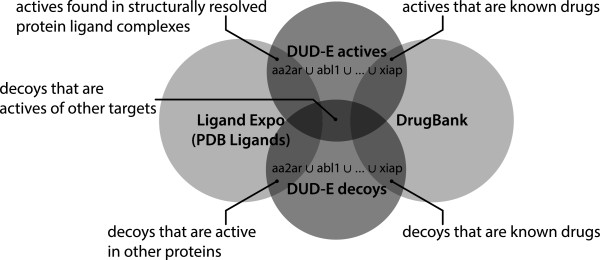
**Verifying existing molecular databases.** Set operations between different DUD‐E subsets for different targets can be used to identify potentially problematic molecules.

Databases like DUD‐E [11,12] are widely used to test and evaluate the performance of docking algorithms. The functionality provided by MONA can be used to simplify verification tasks that are tedious to do manually. In order to validate the new DUD‐E database, we tried to answer the following three questions (see Figure 7): 

● Are any of the actives decoys for other targets?

● Are any of the decoy molecules ligands found in structurally resolved protein‐ligand complexes?

● Are any of the decoy molecules already known drugs?

In order to investigate the first question, one molecule set with actives and one set with decoys was created from the respective files for each individual target. Then, all active sets where united into one set *A* and all decoys where united into one set *D*. The intersection of both sets directly provides the answer to the first question. The resulting set contains 123 molecules (provided in Additional file 1).

To answer the second question, the decoy set *D* has to be intersected with a set containing known ligands from protein‐ligand complexes. The necessary data is provided by LigandExpo [13,14] which offers a SMILES file containing all small molecules from crystal structures in the Protein Data Bank (PDB) [15]. The resulting intersection contains 141 decoys which are ligands of at least one protein in the PDB (provided in Additional file 2).

**Figure 8:**
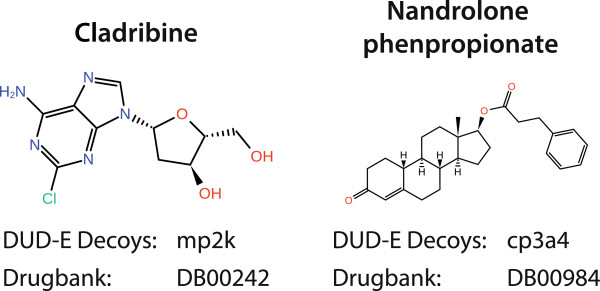
Cladribine and nandrolone phenpropionate are two examples from the 26 molecules that are contained in both DUD‐E decoys and Drugbank.

The third question can be answered in the same way. This time, a substance set of approved drugs from Drugbank [16,17] was used as reference. Drugbank currently lists 1395 molecules registered as drugs. The intersection of these molecules with *D* contains 26 molecules (provided in Additional file 3) each of which is approved as a drug. Most interestingly, the resulting set contains the compound cladribine (see Figure 8), which is known to interact to deoxycytidine kinase and considered as a decoy molecule of mitogen‐activated protein kinase 1. The compound nandrolone phenpropionate is a known substrate to cytochrome P450 19A1 and considered decoy for cytochrome P450 3A4. Although these two molecules might in fact be inactive against their decoy targets, this analysis at least points to critical cases where the decoy status should be further clarified.

Furthermore, it is possible to quickly exploit the data sources like the PDB for seeking alternative targets for all the actives in the DUD‐E dataset. Let *A*_*i*_ be the set of active compounds for each target *i*. The intersections between each *A*_*i*_ and the LigandExpo set results in one set per target containing all compounds for which complex structures are deposited in the PDB. Exporting these sets with all instances taken from LigandExpo results in one file for each target containing other proteins in the PDB with the same ligand. As an example the active flavopiridol for cdk2 was found which also inhibits glycogen phosphorylase (PDB code 1e1y). Note that searching for flavopiridol in the PDB easily gives the same result but with MONA, this search process was performed with all 20289 active molecules of DUD‐E simultaneously without the need for scripting.

It took seven minutes to import all 1.2 million molecules necessary for this scenario into the database and one minute to create all sets in the GUI on an Intel Core i7‐2600 CPU with 3.4 GHz and 8 GB of memory. All individual set operations ran in less than 10 seconds.

### Correctness

All operations provided by MONA depend on the consistent internal representation of molecules and their respective properties. This applies to both the internal chemical model and the operations performed by the underlying database. The consistency of the chemical model concerning the handling of different chemical file formats has already been validated in [7]. Therefore, the validation of MONA was focused on the correctness of the database functionality. This was done by ensuring the following invariants: 

1. Molecules stored in the database are restored exactly as before.

2. Molecule sets can be created and combined with set operations.

3. Different types of filters can be correctly applied to molecule sets.

Storage of molecules in the database is tested by comparing a molecule restored from the database with the original molecule. The order of atoms and bonds may change, but if any valence states or atom coordinates differ the test fails. All molecules passing NAOMI initialization from PubChem Substance (100 M molecules) [18,19] and from emolecules (5 M molecules) [20] can be correctly restored from the database.

**Figure 9:**
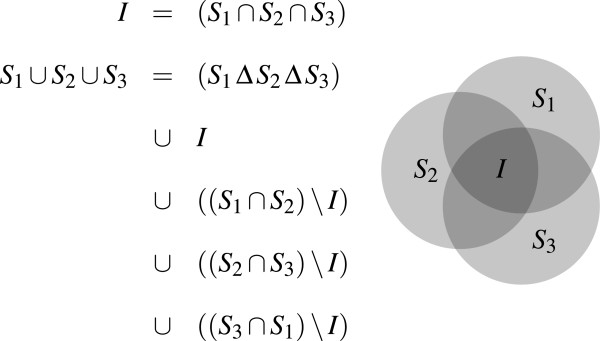
**Testing set operations against each other.** The shown equation was evaluated with three randomly created sets *S*_1_,*S*_2_ and *S*_3_, where *Δ* is the symmetric difference of two sets.

Operations on sets of molecules were tested against each other by verifying that the general equation in Figure 9 holds. Sets *S*_1_,*S*_2_ and *S*_3_ are created by randomly distributing molecules of a test set to one, two or all three sets. Then the union of *S*_1_,*S*_2_ and *S*_3_ must be the same as the union of the symmetric difference (*S*_1_*ΔS*_2_*ΔS*_3_), the intersection of all three sets and all pair‐wise intersections of two sets.

Confirming filter operations was done by comparing results returned by the database against the results retrieved by linearly applying each filter against every molecule in turn.

### Computing time

In order to assess the computing time requirements of MONA, scaling tests for important operations on the database were performed. As most of the operations only consist of database queries the results are highly dependent upon the used database backend. Here, SQLite was used with a page cache of 1 GB. This value was chosen as the best compromise for modern workstations.

All benchmarks were done on a workstation with an Intel Xeon E5630 CPU running at 2.53 GHz and 64 GB of available main memory. A subset of molecules from the PubChem Substances database was used as benchmark set. The molecules in this set were randomly chosen with uniform probability from the whole PubChem Substance database.

**Figure 10:**
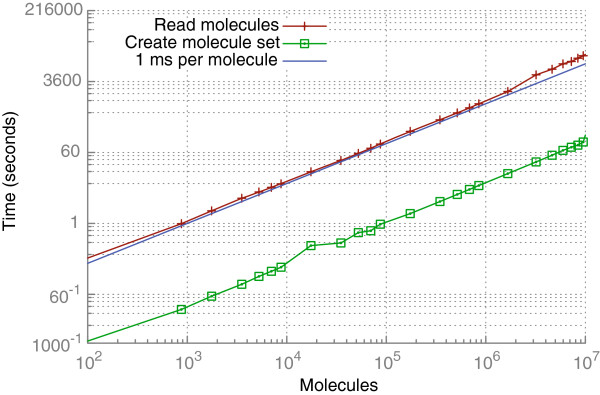
**Requirements for reading molecules from SDF including insertion and duplicate detection.** The red curve shows overall loading time for data files of a particular size (approximately one millisecond per molecule is needed) and the green curve shows the time needed to create a molecule set of this size once the molecules are stored in the database.

Naturally, the size of the database depends linearly on the size of the input. In our case the size of the database corresponds roughly to the size of a compressed SD file of the same compound set. All in all it takes approximately 1000 seconds to read 1 million molecules from SDF (see Figure 10), resulting in a database of size 1 GB, which is much smaller than the respective uncompressed MOL2 or SDF files.

**Figure 11:**
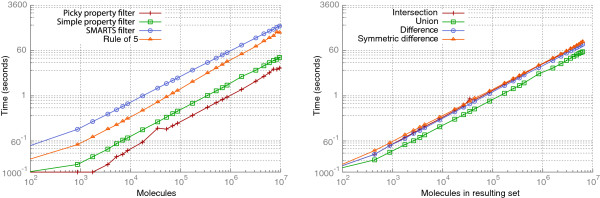
**Computing times of filter and set operations.** All operations clearly show a linear dependence on the number of molecules (for filters, left diagram) or the number of molecules in the resulting set (for set operations, right diagram).

The relative order of run times for different types of filters (see Figure 11) has been discussed in Section “Filtering and visual selection”. Additionally, all filters and set operations do not only depend linearly upon the size of the input set but also on the size of the resulting set. This can be seen when comparing the picky property filter to the simple property filter from Figure 11 as the picky filter has to write considerable less results into a new subset in the database.

In summary, we conclude that MONA is efficient enough to handle sets with up to one million molecules interactively on a current workstation with at least 2 GB of main memory. Therefore, it can be used as a desktop application for most cheminformatics tasks.

## Conclusion

MONA is an intuitive, interactive tool for processing large small‐molecule datasets. It offers functionality to perform many common cheminformatics tasks such as combining datasets, filtering by molecular properties, and visualization using a built‐in 2D engine. Since MONA is based on a robust cheminformatics framework, molecules from common file formats (SMILES, SDF, MOL2) can be handled consistently. The low setup time despite the use of a database makes MONA a reasonable compromise between pipelining tools and molecule database systems. More importantly, MONA offers a different way of working with molecule datasets. Compared to pipelining tools, it supports an interactive and case‐driven process. While chemical databases and pipelining tools are mostly in the hands of cheminformaticians, MONA’s lightweight interface offers chemists an easy way to deal with large compound collections.

We have provided three prototypical scenarios from different fields of applications which emphasize the great versatility of MONA. Various validation procedures show that MONA is internally consistent concerning both the representation of molecules and the database operations. Furthermore, the run times for dataset operations from the benchmarks are sufficient for interactive use in most situations with up to one million molecules.

Since working with datasets is such a central task in cheminformatics there are a lot of potential additional features which could be included in future versions of MONA. We are confident, that MONA’s functionality will be substantially extended over the next year. The main focus will be on the introduction of new types of visualizations for molecular sets with respect to molecular similarity and molecular scaffolds. The current version can be downloaded at http://www.zbh.uni‐hamburg.de/mona. It is available free of charge for academic use.

## Competing interests

The authors declare that they have no competing interests.

## Authors’ contributions

M.H. and S.U. developed the algorithmic concepts behind MONA, implemented the software and tested it. I.G. and S.H. participated in the user interface design and performed initial tests. M.R. initiated the development and supervised the project. All authors read and approved the final manuscript.

## Supplementary Material

Additional file 1 The file contains all molecules from the intersection between a set containing all DUD‐E decoys and a set containing all DUD‐E actives (123 molecules). Click here for file

Additional file 2 The file contains all molecules from the intersection between a set containing all DUD‐E decoys and a set containing all LigandExpo molecules (141 molecules). Click here for file

Additional file 3 The file contains all molecules from the intersection between a set containing all DUD‐E decoys and a set containing all DrugBank molecules (23 molecules). Click here for file
